# Identification of Key Factors Involved in the Biosorption of Patulin by Inactivated Lactic Acid Bacteria (LAB) Cells

**DOI:** 10.1371/journal.pone.0143431

**Published:** 2015-11-18

**Authors:** Ling Wang, Zhouli Wang, Yahong Yuan, Rui Cai, Chen Niu, Tianli Yue

**Affiliations:** College of Food Science and Engineering, Northwest A & F University, Yangling, Shaanxi, 712100, China; Agricultural University of Athens, GREECE

## Abstract

The purpose of this study was to identify the key factors involved in patulin adsorption by heat-inactivated lactic acid bacteria (LAB) cells. For preventing bacterial contamination, a sterilization process was involved in the adsorption process. The effects of various physical, chemical, and enzymatic pre-treatments, simultaneous treatments, and post-treatments on the patulin adsorption performances of six LAB strains were evaluated. The pre-treated cells were characterized by scanning electron microscopy (SEM). Results showed that the removal of patulin by viable cells was mainly based on adsorption or degradation, depending on the specific strain. The adsorption abilities were widely increased by NaOH and esterification pre-treatments, and reduced by trypsin, lipase, iodate, and periodate pre-treatments. Additionally, the adsorption abilities were almost maintained at pH 2.2–4.0, and enhanced significantly at pH 4.0–6.0. The effects of sodium and magnesium ions on the adsorption abilities at pH 4 were slight and strain-specific. A lower proportion of patulin was released from the strain with higher adsorption ability. Analyses revealed that the physical structure of peptidoglycan was not a principal factor. Vicinal OH and carboxyl groups were not involved in patulin adsorption, while alkaline amino acids, thiol and ester compounds were important for patulin adsorption. Additionally, besides hydrophobic interaction, electrostatic interaction also participated in patulin adsorption, which was enhanced with the increase in pH (4.0–6.0).

## Introduction

Patulin is a mycotoxin biosynthesized by several fungal species belonging to the genera *Penicillium*, *Aspergillus* and *Byssochlamys* [1]. Patulin can contaminate various fruits, especially in apple and apple products worldwide, and has been shown to have detrimental effects on the health of humans and animals [2]. Once activated, patulin may exhibit carcinogenic, mutagenic, teratogenic and immunosuppressive effects [3]. In this regard, Food and Agriculture Organization of the United Nations (FAO), World Health Organization (WHO) and European Union (EU) have established the provisional maximum tolerable daily intake of patulin, and the maximum levels of patulin in fruit products, respectively [4].

Attempts have been made to develop methods either to remove patulin from contaminated foods and feeds or to degrade the present toxin into less toxic compounds [5]. However, these methods are expensive and impractical for some regulatory limits [6], and can diminish the quality of fruit juices [5]. An alternative strategy is the use of inactivated micro-organisms to adsorb patulin at high efficiency, while having minimal effects on juice qualities [7–11]. Since many lactic acid bacteria (LAB) strains are food-grade micro-organisms [12], the use of inactivated LAB cells is a promising approach to remove patulin toxin. According to Hatab *et al*. [11], some inactivated LAB strains can be used for removing patulin from apple juice, without posing any negative impact on the quality parameters of apple juice.

Currently, our research team has carried out much work on the adsorption mechanism of patulin by inactivated cells and found that some carbohydrate and protein moieties and hydrophobic interaction were important for adsorption of patulin [4,11,13,14]. However the adsorption mechanism of patulin by inactivated cells remains unclear as some limitations still exist. Firstly, the extensive overlap of diagnostic infrared spectroscopy (IR) bands restricts the application of Fourier transform infrared spectroscopy (FTIR) in studies due to the complexity of cell composition. Some unnecessary groups would be misjudged as functional groups and some other functional groups would be missed. Secondly, as only a strain of yeast was used to study the effects of chemical and enzymatic pre-treatments on patulin adsorption, the adsorption mechanism found in the investigation may not be broadly applicable [13]. Thirdly, although it is well documented that the solution pH is an important parameter affecting biosorption process, the relative contribution of bacterial surface charge to patulin adsorption is not clearly understood. Fourthly, little attention has been paid to the stability of washed cell-patulin complexes, the understanding of which may help better reveal the mechanism. Finally, any potential bacterial contamination in the adsorption process needs to be avoided.

Focusing on the above-mentioned issues, this study attempted to investigate the universal and key functional groups, compounds, and interactions involved in the adsorption of patulin by heat-inactivated LAB cells. Additionally, our secondary goal was to select pre-treatment methods which could more widely promote the patulin adsorption ability of heat-inactivated LAB cells.

## Materials and Methods

**Fig 1:**
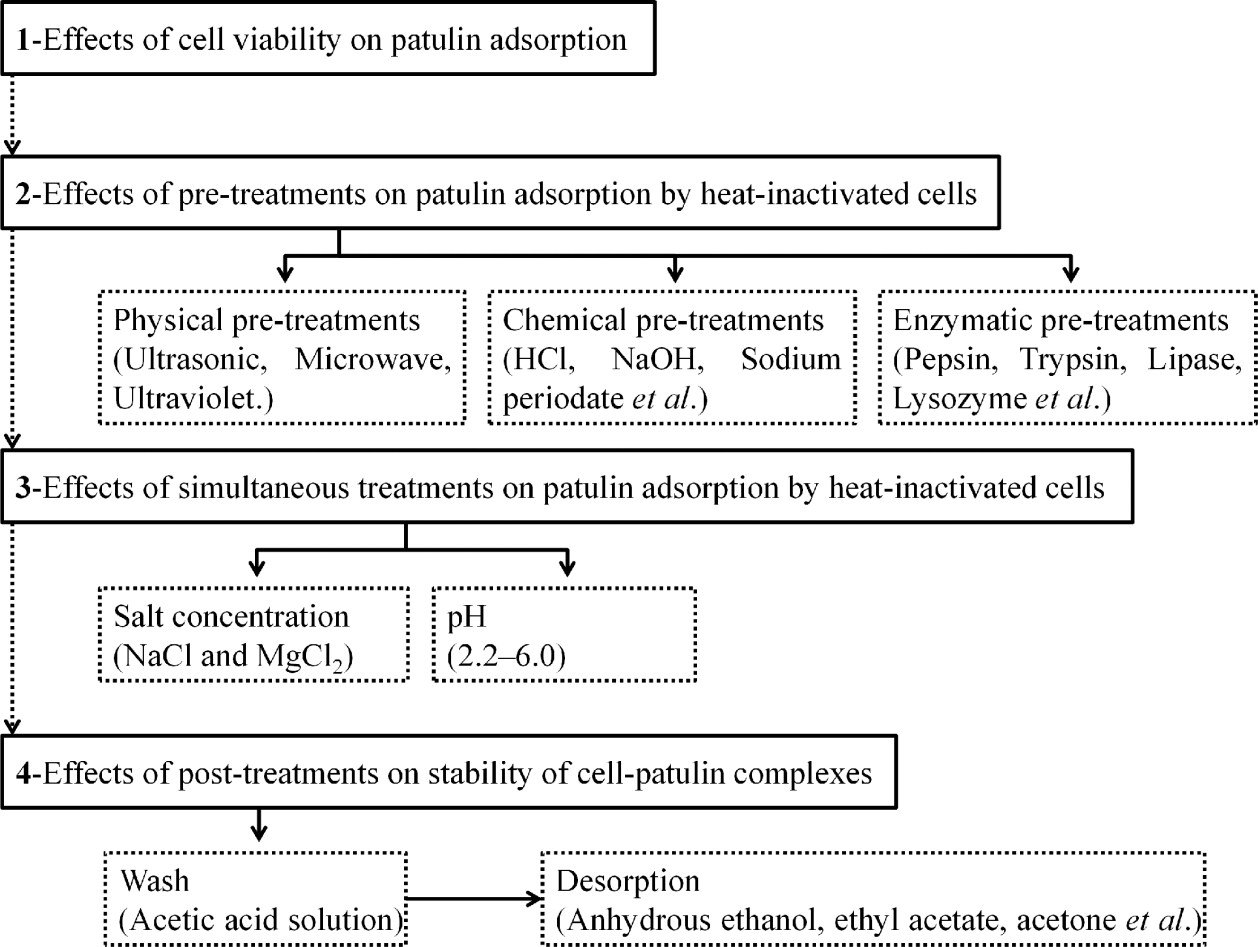
Flow diagram outlining the experimental design.

In this study, the effects of various pre-treatments, simultaneous treatments, and post-treatments on the patulin adsorption performances of six LAB strains were evaluated. The experiments were done following the flow diagram described in **Fig 1**and explained in detail below.

### Chemicals and equipment

Standard patulin and acetonitrile (HPLC grade) were purchased from Sigma-Aldrich (St Louis, MO, USA). Pronase E (Protease from *Streptomyces griseus*, cat. no., P0652), trypsin (Trypsin from bovine pancreas, cat. no., T1426), pepsin (Pepsin from porcine gastric mucosa, cat. no., P7012), lipase (Lipase from porcine pancreas, cat. no., L3126), and lysozyme (Lysozyme from chicken egg white, cat. no., L6876) were purchased from Sigma-Aldrich. All other chemicals used in the experiments were obtained from local chemical reagent company (Yangling, China).

*Lactobacillus curvatus* 21019 (LC-21019), *Lactobacillus rhamnosus* 6133 (LR-6133), *Lactobacillus rhamnosus* 6224 (LR-6224), *Lactobacillus brevis* 20023 (LB-20023), *Enterococcus faecium* 20420 (EF-20420) and *Enterococcus faecium* 21605 (EF-21605) were purchased from China Center of Industrial Culture Collection (CICC). The culture conditions and preparation of heat-inactivated LAB cells powder were described in detail elsewhere [4]. The powdered bacterial cells were used for pre-treatments and adsorptions.

An ultraviolet (UV) lamp (253.7 nm, 30 Watts, ZW30S19W, Jiangyin Feiyang Equipment Co., Ltd., China), a household microwave oven (G70F20N2L-DG (W0), Guangdong Galanz Enterprise Group Co. Ltd., China, 700 watts, 2,450 MHz), a ultrasonic cell disintegrator (JY92-IIDN, Ningbo Scientz Biotechnology Co., Ltd., China) and a scanning electron microscope (SEM; Leo Zeiss 435 VP SEM, Oberkochen, Germany) were used.

### Patulin solution preparation and adsorption assay

A standard stock solution of patulin at a concentration of 200 mg/L was prepared in ethyl acetate and stored at -40°C. A working solution of patulin at a concentration of 2 mg/L or 4 mg/L was prepared by evaporating the ethyl acetate at 40°C in a water bath to dryness, then immediately dissolving the residue in acetic acid solution (pH 4.0). These solutions were kept at 4°C, renewed weekly, and brought to room temperature before use.

To investigate the effect of cell viability on patulin adsorption ability, the acetic acid solution (pH 4.0) and patulin working solution (4 mg/L) were each sterilized by passing them through 0.22 micron bacteria-retentive filters. After the bacterial cultivation, part of bacterial cultures was divided into two aliquots and centrifuged respectively. Pelleted bacteria cells obtained from one aliquot were killed at 121°C for 20 min and washed at least three times with sterile acetic acid solution (pH 4.0). Pelleted bacteria cells obtained from the second aliquot were processed similarly without the heat treatment. Pelleted viable or heat-inactivated LAB cells were mixed with 1 mL patulin working solution (sterile, 4 mg/L) and were added to sterile acetic acid solution to final 2 mL, respectively. The whole process was carried out under aseptic conditions in order to prevent the bacterial contamination. The reaction mixtures were shaken on an orbital shaker at 150 rpm, 37°C for 48 h.

To investigate the effects of pre-treatments on adsorption of patulin by heat-inactivated LAB cells, 0.01 g of heat-inactivated LAB cells materials (the pre-treated or untreated heat-inactivated LAB cells) were suspended in 2 mL of patulin working solution (2 mg/L). In order to prevent bacterial contamination, tubes containing suspensions were heated in a thermostatic water bath operating at 97°C for about 2 minutes. Then these samples were cooled to room temperature and incubated at 37°C, 150 rpm for 48 h.

To investigate the effect of salt concentration on adsorption of patulin by heat-inactivated LAB cells, the ionic strength of patulin working solution (2 mg/L) was adjusted by adding NaCl or MgCl_2_ (0.1, 0.4, 0.7, or 1.0 mol/L, respectively); to investigate the effect of pH, patulin solutions (4 mg/L) were prepared by immediately dissolving patulin in McIlvaine’s buffer (pH 2.2, 3, 4, 5, and 6, respectively) after evaporating the ethyl acetate at 40°C in a water bath to complete dryness. These solutions were prepared immediately before use. The patulin solution of varying ionic strength or pH (2 mL) was added directly to 0.01 g untreated heat-inactivated LAB cells. Next, the sterilization and patulin adsorption were performed as described above.

At the end of each incubation period, suspensions were centrifuged and supernatants were collected for analysis of patulin content. All assays were performed in triplicate. Negative and positive controls were also performed.

### Pre-treatments of heat-inactivated LAB cells powder

As listed in **Table 1**, for microwave and UV treatments, heat-inactivated LAB cells (0.15 g dry weight) were treated. For ultrasonic treatment, a 50 mL heat-inactivated LAB cells suspension (2 g/L) was prepared in distilled water and disintegrated in an ice-water bath by the ultrasonic cell disintegrator. For chemical and enzymatic modification, 0.15 g heat-inactivated LAB cells were incubated in different reagents (37°C, 150 rpm) over differing durations. After each pre-treatment was performed, suspensions were centrifuged. Treated bacterial cells were washed at least four times with 9-mL distilled water until the pH of the wash solution reached neutral range (about pH 6.8) and then freeze-dried. Dried biomass was ground and subjected to the adsorption assay. Pre-treated and untreated heat-inactivated bacterial cells were observed and photographed with a scanning electron microscope, operating at 15 kV.

**Table 1:** Pre-treatment methods applied to heat-inactivated LAB cells.

Type	Pre-treatment	Duration
*A*	No treatment	–
*B1*	Ultrasonic, performed at 300W, every other 20 s for 15 min	15 min
*B2*	Ultrasonic, performed at 500W, every other 20 s for 15 min	15 min
*B3*	Ultrasonic, performed at 700W, every other 20 s for 15 min	15 min
*C1*	Microwave(700 W)	2 min
*C2*	Microwave(700 W)	5 min
*C3*	Microwave(700 W)	8 min
*D1*	Ultraviolet (100 μW/cm^2^)	1 h
*D2*	Ultraviolet (100 μW/cm^2^)	2 h
*D3*	Ultraviolet (100 μW/cm^2^)	3 h
*E*	HCl (2 mol/L, 9 mL)	3 h
*F*	NaOH (0.1 mol/L, 9 mL)	3 h
*G*	Formaldehyde and formic acid (V:V = 1:2, 9 mL)	6.5 h
*H*	Acetone (9 mL)	6.5 h
*I*	Methanol and concentrated sulfuric acid (V:V = 99:1, 9 mL)	48 h
*J*	Urea (8 mol/L, 9 mL)	3 h
*k'* ^a^ ^,^ ^c^	Acetate buffer (pH 4.5, 0.01 mol/L, 9 mL)	3 h
*k″* ^b^ ^,^ ^c^	Sodium iodate (0.01 mol/L prepared in acetate buffer, pH 4.5, 9 mL)	3 h
*K* ^c^	Sodium periodate (0.01 mol/L prepared in acetate buffer, pH 4.5, 9 mL)	3 h
*l'* ^a^ ^,^ ^d^	HCl (0.2%~0.4%, pH 2, 9 mL)	3 h
*L* ^d^	Pepsin (1 mg/mL prepared in HCl (0.2%~0.4%, pH 2), 9 mL)	3 h
*m'/n'/o'* ^a^ ^,^ ^d^	Phosphate buffer saline (PBS) (pH 7.6, 0.01 mol/L, 9 mL)	3 h
*M* ^d^	Pronase E (1 mg/mL prepared in PBS, pH 7.6, 9 mL)	3 h
*N* ^d^	Trypsin (1 mg/mL prepared in PBS, pH 7.6, 9 mL)	3 h
*O* ^d^	Lipase (1 mg/mL prepared in PBS, pH 7.6, 9 mL)	3 h
*p'* ^a^ ^,^ ^d^	Phosphate buffer saline (PBS) (pH 6.5, 0.01 mol/L, 9 mL)	3 h
*P* ^d^	Lysozyme (1 mg/mL prepared in PBS, pH 6.5, 9 mL)	3 h

^a^ *k′* as a solvent control pre-treatment of *k″* and *K*

*l'* as a solvent control pre-treatment of *L*

*m'/n'/o'* as a solvent control pre-treatment of *M*, *N*, and *O*

*p′* as a solvent control pre-treatment of *P*.

^b^ *k″* as a control pre-treatment of *K*.

^c^ Containers were wrapped in foil to protect the solutions from light.

^d^ Solutions were sterilized through 0.22-μm filters and used right after they were ready.

### Post-treatments

For each strain, 0.01 g of untreated heat-inactivated LAB cells was suspended in 2 mL of patulin working solution (4 mg/L). After heating in a water bath, cell-patulin suspensions were incubated at 37°C, 150 rpm for 72 h. After the 72-h incubation, a supernatant sample (200 μL) was collected to determine the patulin content, and the rest of the supernatant was discarded. Cell-patulin complexes obtained as previously described were washed 3 times with 2 mL acetic acid solution and then freeze-dried. Dried cell-patulin complexes were subsequently extracted with 2 mL anhydrous ethanol, ethyl acetate, acetone, or acetic acid solution (37°C, 150 rpm for 48 h). Supernatants from anhydrous ethanol, ethyl acetate or acetone extraction process were collected, dried, dissolved (2 mL of acetic acid solution) and analyzed by high-performance liquid chromatography (HPLC). Supernatants from acetic acid solution extraction were collected and directly analyzed by HPLC. The percentage of patulin released by washed cell-patulin complexes (*Y*_*R*_) was determined by the following formula:

$$Y_{R}=R/A\times 100$$ (1)

Where *R* was the amount of patulin released and *A* was the amount of patulin adsorbed.

### Detection and quantification of patulin

Reverse-phase HPLC (Shimadzu LC-20AD pump, CTO-20A column oven, and SPD-M20A detector; Shimadzu Scientific Instruments, Columbia, MD) was used to quantitate adsorbed patulin. The percentage of patulin adsorbed to the bacterial cells was calculated by the following formula:

$$Y_{A}=\left(A/T\right)\times 100$$ (2)

Where *Y*_*A*_ was the adsorbed rate of patulin, *A* was the amount of patulin adsorbed and *T* was the total amount of patulin in positive control.

### Statistical analysis

All experiments were performed in triplicate, and the data were presented as means ± standard deviation (SD). SAS procedure (SAS version 9.1, SAS Inst. Inc., Cary, N.C., U.S.A.) was used to evaluate any significant differences between samples. Statistical significance was set at *P* < 0.05.

## Results

### Effects of cell viability on patulin adsorption

**Fig 2:**
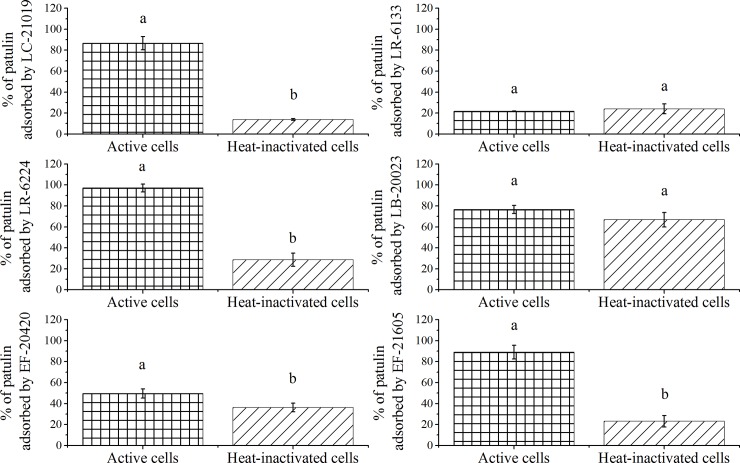
Effects of cell viability on patulin-elimination abilities of LAB cells. Bars represent means of triplicate assays and error bars represent SD. Values of bars labeled by different lowercase letters were significantly different (*P* < 0.05).

To determine whether the viability of the bacteria affected their detoxification properties, comparative experiments with viable and heat-inactivated cells were conducted. The results were shown in **Fig 2**. Both viable and heat-inactivated cells of the strains showed the ability to reduce patulin. For LC-21019, LR-6224, EF-20420, and EF-21605 strains, patulin was more efficiently decreased by viable cells than heat-inactivated cells. In contrast, for LR-6133 and LB-20023 strains, there was no significant difference between the elimination ability of viable and inactivated cells.

### Effects of pre-treatments on patulin adsorption

**Fig 3:**
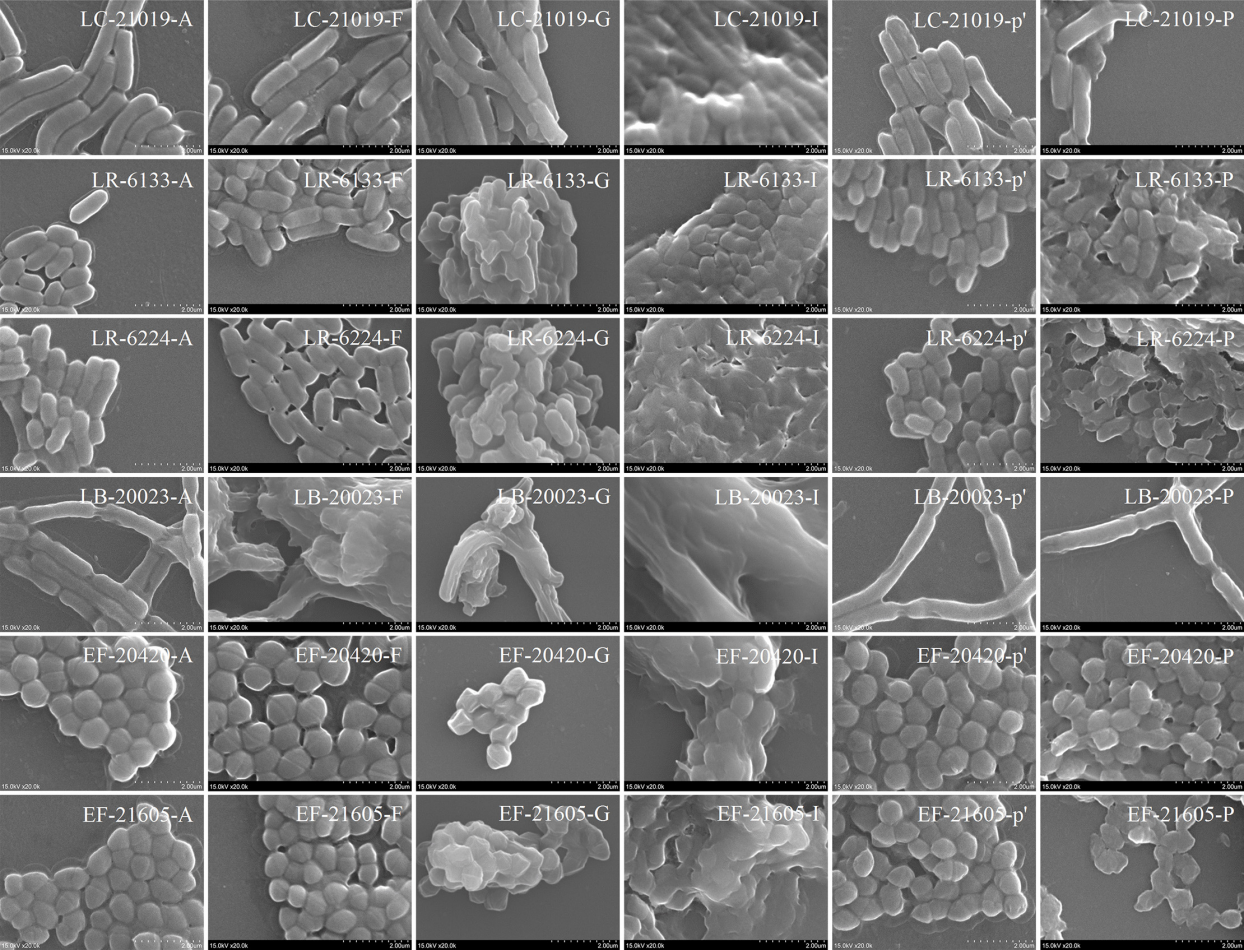
Scanning electron micrographs of untreated heat-inactivated LAB cells (Type *A*) and the heat-inactivated cells pre-treated with NaOH (Type *F*), formaldehyde (Type *G*), methanol (Type *I*), lysozyme (Type *P*) and the solvent control (Type *p'*). Magnification 20,000×. Bar, 2.0 μm.

The effects of pre-treatments on the surface morphologies of heat-inactivated cells were strain specific. Representative SEM micrographs are shown in **Fig 3**. Untreated heat-inactivated LAB cells (Type *A*) showed the classical cell shape of *bacillus* or *coccus* with a regular and smooth surface. It was clear that NaOH pre-treated cells (Type *F*) of LB-20023 showed significant damage of the surface morphology, which was different from the other strains that had little changes. For LR-6133, LR-6224, EF-20420, and EF-21605 strains, the lysozyme pre-treated cells (Type *P*) were significantly changed and damaged compared with the solvent control (Type *p'*); but for LC-21019 and LB-20023 strains, the damages were more moderate without the formation of broken shreds. By contrast, for all strains tested here, some folds appeared on the surfaces of cells and without obvious disrepairs after pre-treatment with formaldehyde (Type *G*); the extent of the aggregation of the methanol pre-treated cells (Type *I*) was higher than that of untreated cells, and obvious adhesions appeared between pre-treated cells.

**Fig 4:**
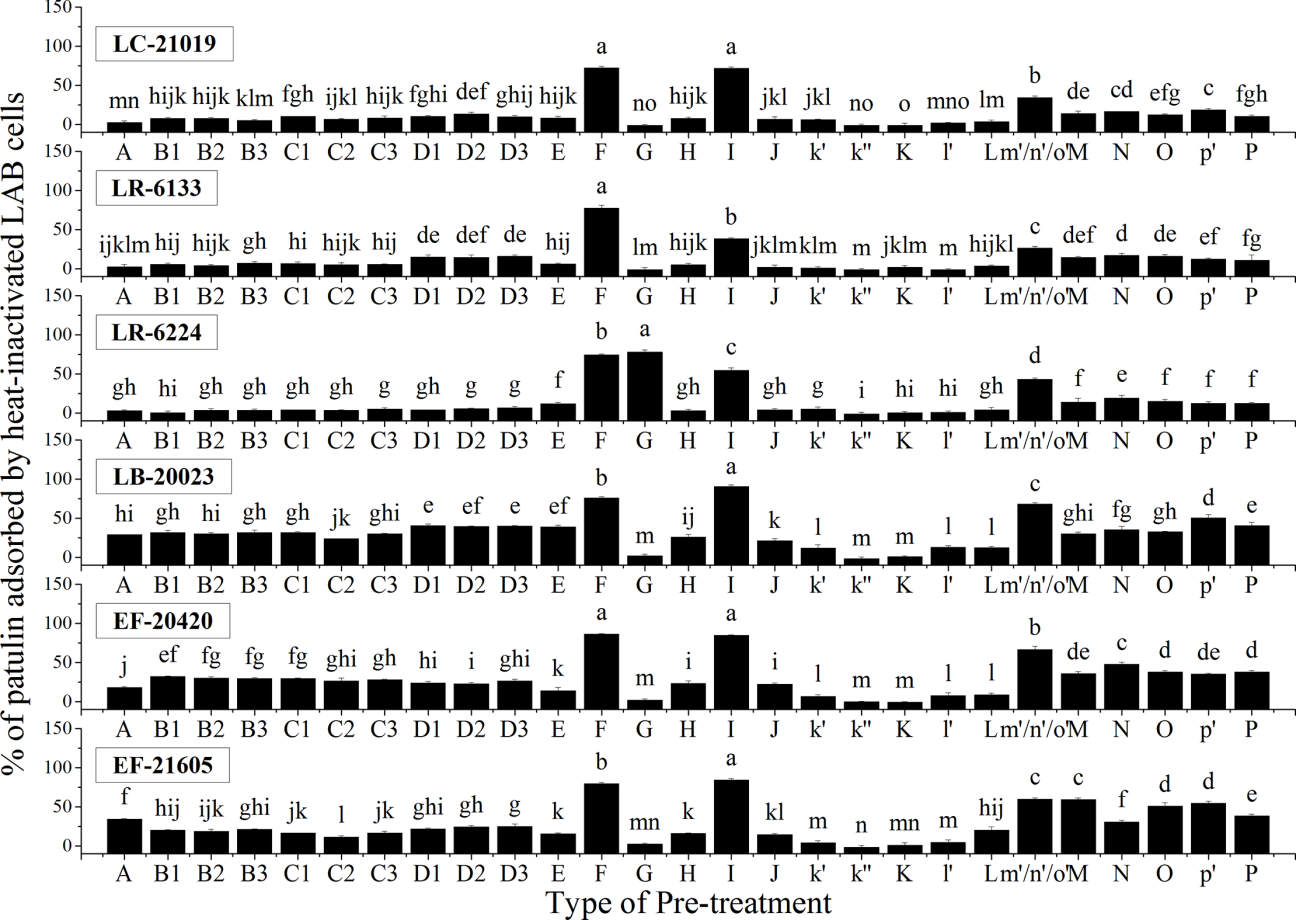
Effects of pre-treatments on patulin adsorption abilities of heat-inactivated LAB cells. Bars represent means of triplicate assays and error bars represent SD. Values of bars labeled by different lowercase letters were significantly different (*P* < 0.05).

The effects of pre-treatments on the adsorption of patulin are shown in **Fig 4**. For physical pre-treatments, ultrasonic (Types *B1*, *B2*, and *B3*), microwave (Types *C1*, *C2*, and *C3*) and UV (Types *D1*, *D2*, and *D3*) pre-treatments significantly improved the ability of EF-20420 strain to adsorb patulin. The effects for LC-21019 were similar to EF-20420, but the ultrasonic pre-treatment Type *B3* almost maintained the adsorption of patulin (*P* > 0.05). On the contrary, all of these physical pre-treatments significantly reduced the ability of EF-21605 strain to adsorb patulin. Additionally, ultrasonic, microwave, and UV pre-treatments did not significantly alter the adsorption ability of LR-6224 towards patulin. As for the LR-6133 and LB-20023 strains, almost all the ultrasonic and microwave pre-treatments had no effect on adsorption except Type *B3* for LR-6133 and Type *C2* for LB-20023. In contrast, UV pre-treatments (Types *D1*, *D2*, and *D3*) significantly improved the abilities of LR-6133 and LB-20023 strains to adsorb patulin.

Similarly, positive and negative impacts of most chemical and enzymatic pre-treatments on the adsorption of patulin were varied within bacterial species. In contrast, for all strains tested here, pre-treatments with NaOH (Type *F*) or methanol (Type *I*) resulted in great increases in patulin adsorption; while trypsin (Type *N*), lipase (Type *O*), sodium iodate (Type *k″*), and sodium periodate (Type *K*) pre-treatments widely reduced the patulin adsorption compared with the corresponding controls.

### Effects of simultaneous treatments on patulin adsorption

**Fig 5:**
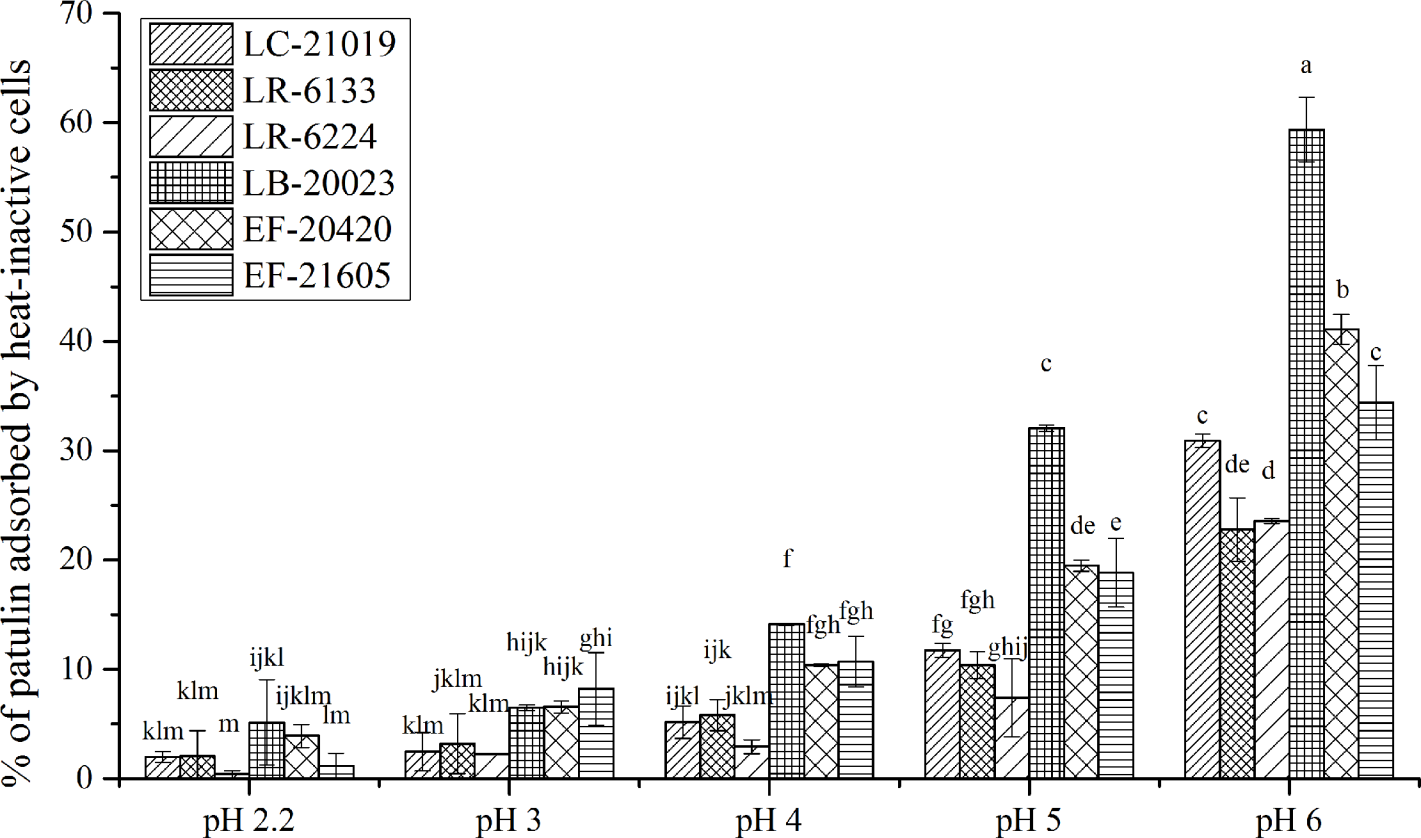
Effects of pH on patulin adsorption abilities of heat-inactivated LAB cells. Bars represent means of triplicate assays and error bars represent SD. Values of bars labeled by different lowercase letters were significantly different (*P* < 0.05).

The effects of pH on patulin adsorption by heat-inactivated LAB cells were shown in **Fig 5**. As depicted in the figure, there was a general trend: the adsorption abilities were almost maintained at pH ≤ 4.0, whereas, when the pH value was raised from 4.0 to 6.0, the adsorption abilities were enhanced significantly. But in some specific situations these strains exhibited a different pattern. For LC-21019 or LR-6133 strains, adsorption abilities did not differ significantly (*P* > 0.05) at pH 2.2–4.0. For LR-6224, besides the similar situation at pH 2.2–4.0, the adsorption ability remained more or less constant at pH 4.0–5.0 (*P* > 0.05). For LB-20023, pH only had no significant effect on patulin adsorption at pH values between 2.2 and 3.0 (*P* > 0.05). For EF-20420, when the pH increased from 2.2 to 4.0, the adsorption ability was almost maintained with a very small extent increase (pH 3–4). Specially, for EF-21605, an increase in patulin adsorption ability was obtained as the pH increased from 2.2 to 3.0 (*P* < 0.05), then an almost constant adsorption ability was showed at pH 3.0–4.0 (*P* > 0.05). As the pH was further increased, the adsorption ability of EF-21605 increased again.

**Fig 6:**
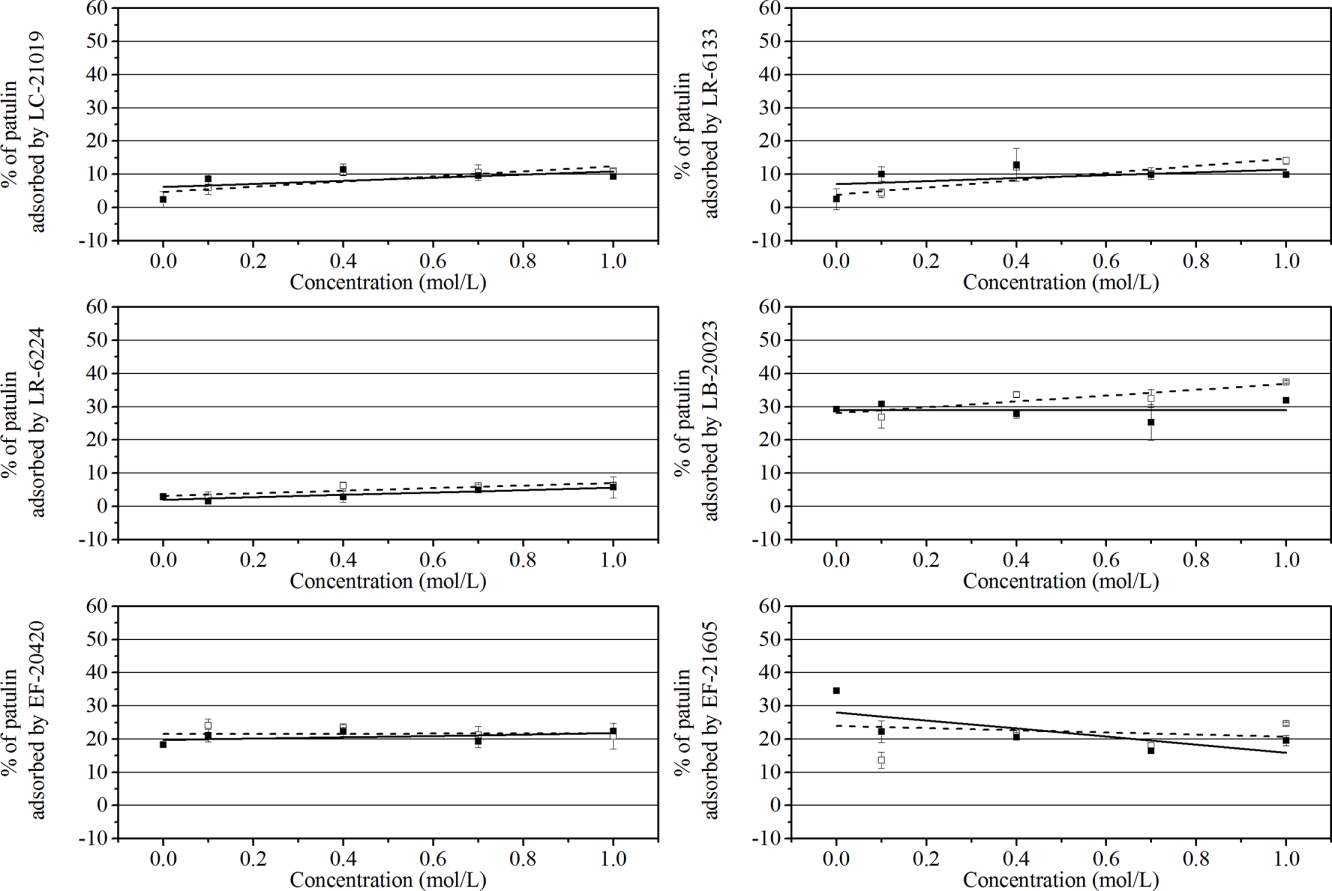
Effects of different concentrations of NaCl (□) and MgCl_2_ (■) on patulin adsorption abilities of heat-inactivated LAB cells. Data shown are the mean ± SD. of triplicates.

The effects of different concentrations of NaCl and MgCl_2_ on patulin adsorption by heat-inactivated LAB cells are shown in **Fig 6**. For LC-21019, LR-6133, and LB-20023 strains, metal ions were propitious for patulin adsorption (*P* < 0.05) with slightly more marked in the presence of NaCl than in the presence of MgCl_2_. In contrast, for LR-6224 and EF-20420 strains, metal ions resulted in no significant changes in adsorption (*P* > 0.05). On the contrary, for EF-21605, the presence of NaCl or MgCl_2_ decreased the adsorption percentage of patulin, but the presence of NaCl was reduced to a lesser extent than the presence of MgCl_2_. These studies showed that the effects of the types and concentrations of metal ions on the patulin adsorption at pH 4 were slight and strain specific.

### Effects of post-treatments on stability of cell-patulin complexes

The stability of cell-patulin complexes was studied by the application of repeated washes with acetic acid solution and then extraction with anhydrous ethanol, ethyl acetate, acetone, or acetic acid solution.

**Fig 7:**
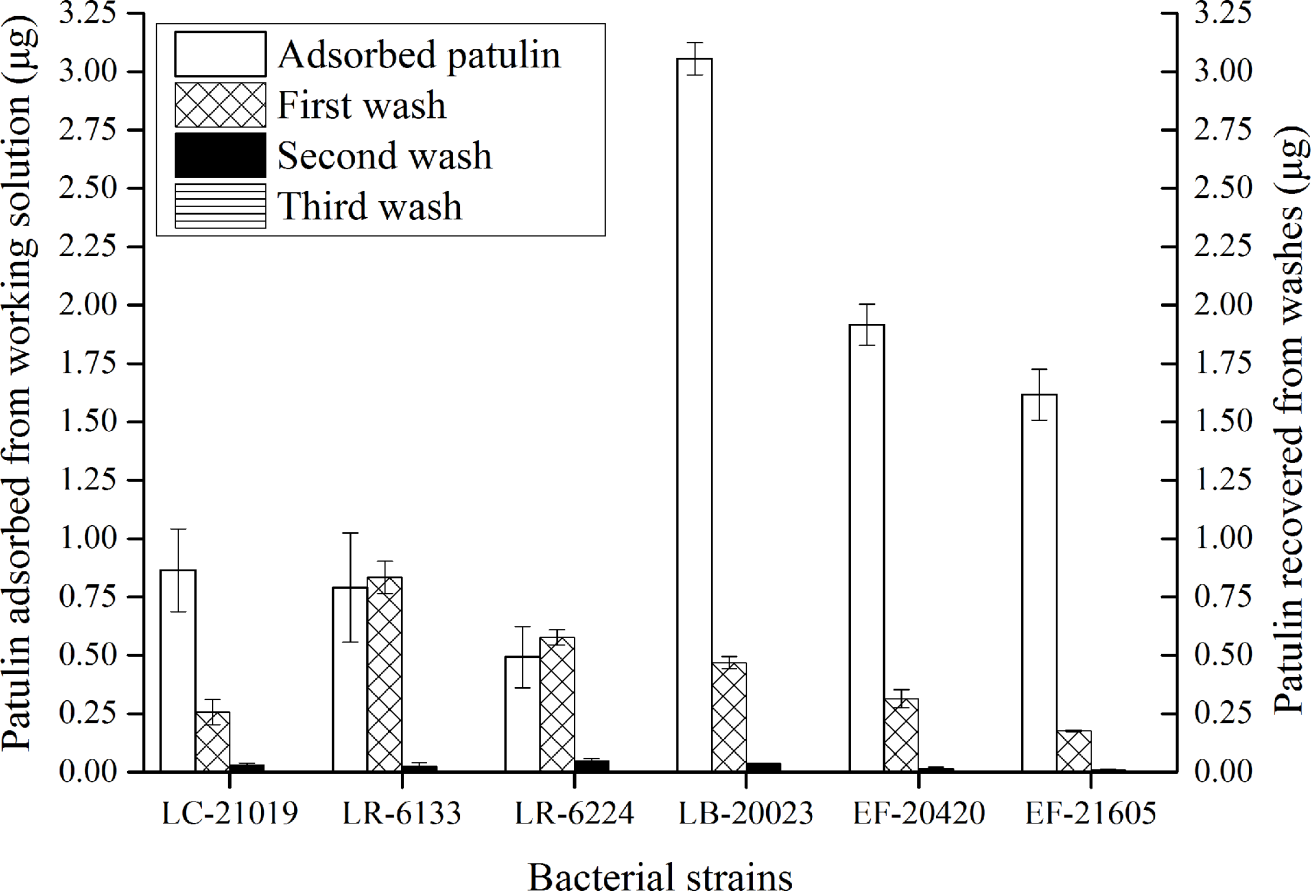
The amounts of patulin recovered from washes. The adsorbed amount of patulin was determined after incubating untreated heat-inactivated cells (0.01 g) with a patulin solution (2 mL, 4 mg/L) for 72 h at 37°C. The cell-patulin complexes were subjected to three washes with 2 mL of acetic acid solution, and the amount of recovered patulin was quantitated. Bars represent means of triplicate assays and error bars represent SD.

The cell-patulin complexes were rinsed with acetic acid solution to remove residual patulin. Amounts of patulin in acetic acid solution after each wash were shown in **Fig 7**, which depicts representative results for the six LAB strains. Almost all the residual patulin was washed away after two wash cycles. For the first two washes, it is worth noting that although volumes of residual patulin for each strain were similar, amounts of patulin in acetic acid solution after each wash were apparently different between strains. We found that the patulin in the wash solution included not only the residual patulin, but also the patulin which was desorbed from cell-patulin complexes. Through the comparison between the amount of patulin adsorbed and recovered (including patulin residual and patulin desorbed), LR-6224 showed the highest desorption abilities, either in the first wash or in the second wash.

**Fig 8:**
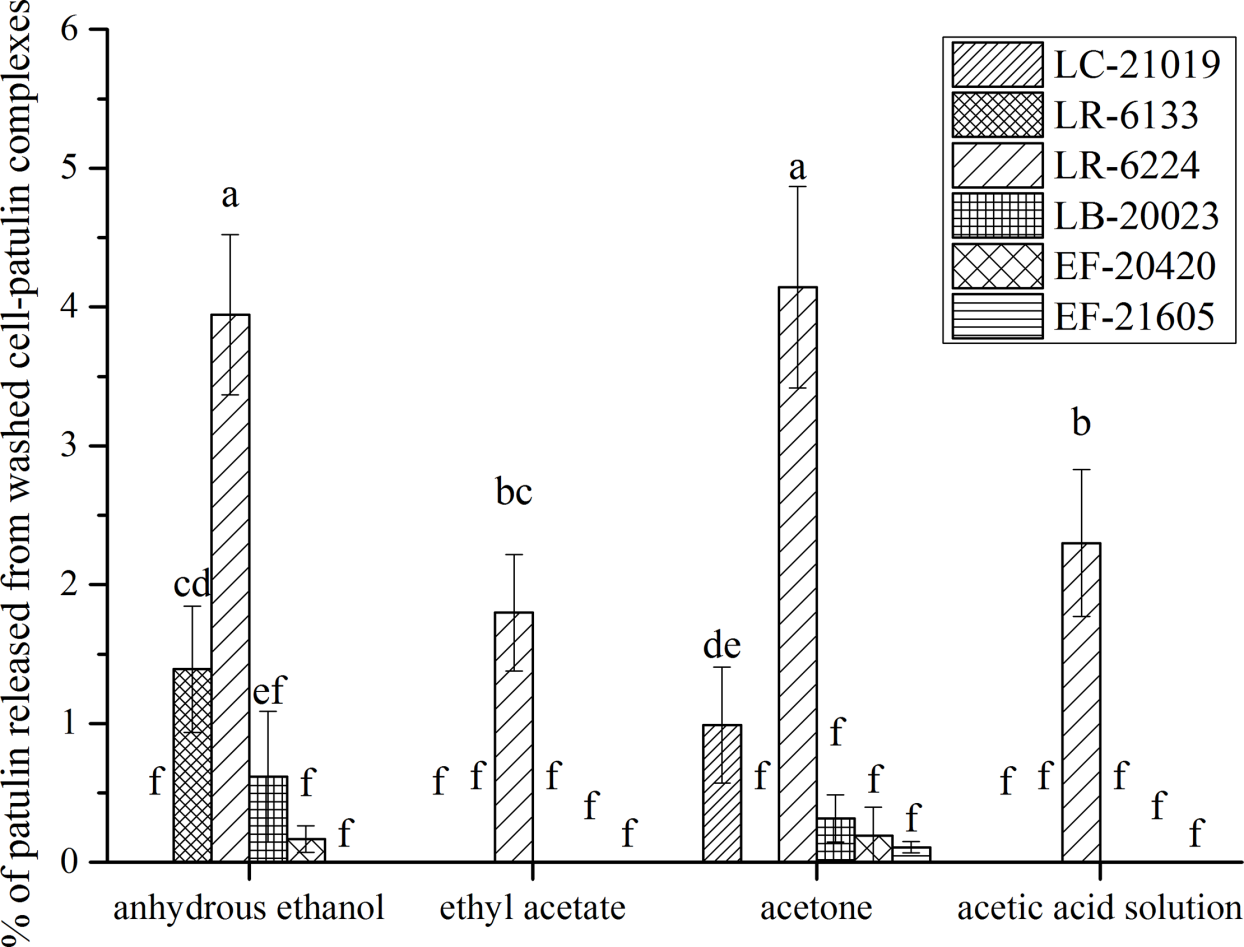
Effects of different solvents on percentages of patulin released from washed cell-patulin complexes. Bars represent means of triplicate assays and error bars represent SD. Values of bars labeled by different lowercase letters were significantly different (*P* < 0.05).

The stability of the washed cell-patulin complexes was studied by the application of incubation with different solutions (**Fig 8**). A small amount of patulin (< 5%) was released from the washed cell-patulin complexes for different eluent treatments. It appeared that anhydrous ethanol and acetone had better desorption efficiency among the eluents tested. For all eluents used in this study, LR-6224 had the best desorption efficiency among the six LAB strains. Essentially, desorption was almost invisible for LB-20023, EF-20420 or EF-21605 (*P* < 0.05) in all the eluents. For LC-21019, desorption of patulin was invisible in anhydrous ethanol, ethyl acetate or acetic acid solution, whereas the desorption efficiency increased when acetone was used as a desorption agent. In contrast, the washed cell-patulin complex of LR-6133 only released patulin in anhydrous ethanol. It was observed that the efficiency of desorption for the strains tested did not increase with the decline of polarity of eluents. This indicated that the stability of the cells-patulin complex depended not only on the polarity of eluent, but also on the strain and other effects of the eluent.

## Discussion

Our results showed that the elimination ability of heat-inactivated cells was equivalent (for LR-6133 and LB-20023) to, or lower (for LC-21019, LR-6224, EF-20420, and EF-21605) than that of viable cells. The results are partly different from those of other authors who only reported one of the two phenomena [13,15–18]. More strikingly, Hawar *et al*. [16] described that patulin elimination was completely halted after subjecting the viable cells to a 121°C autoclave treatment. Although the research found that for several special LAB strains the elimination abilities of viable cells was more effective, inactivated cells could be applied more broadly since using such biomass had almost no impact on qualities of end products [4,11]. We thus concluded that bacterial viability was not a prerequisite for patulin elimination. However, as patulin is biodegradable and can be biosynthesized by several fungal species, the bacterial contamination existing in the adsorption process should be avoided.

Based on the results of observations and experiments and relative literature (**Table 2**and **Table 3**), the effects of all the pre-treatments were analyzed and compared for identifying key and broadly available adsorption mechanisms of patulin and the positive pre-treatment methods.

**Table 2:** Comprehensive effects of pre-treatments on microbial cells.

Pre-treatment	Effect
Ultrasound	Physical (surface resonance), mechanical (shear forces) and chemical (hydrogen ions, hydroxide radicals and H_2_O_2_) effects arising from acoustic cavitation [28].
Microwave	Heat damages and changes of secondary and tertiary structure of proteins by microwave electric field [29].
Ultraviolet (253.7 nm)	Formation of cyclobutyl-type dimers, pyrimidine adducts and DNA-protein crosslinks [30].
Acid	Decrease of cell wall thickness, reduction of cross-links, and increase of pore size by breaking the glycosidic linkages in polysaccharides and the amide linkages in peptides or proteins [31].
NaOH	Removal of compounds which coat porous surface, rupture of cell membrane [32] and neutralization of acidic chemical groups.
Acetone	Solubilization of non-covalently bound apolar glycolipids [23] and leakage of cytoplasmic constituents [14].
Urea	Anti-hydrophobic agent [13]. Release of non-covalently bound proteins [33].

**Table 3:** Major effects of pre-treatments on special constituents of microbial cells.

Pre-treatment	Effect
Proteins and amino groups	
Formaldehyde	Methylation of amino groups, guanidino group [34] and aliphatic amines [35]. Cyclization [36].
Pepsin	Peptide bonds comprising amino of aromatic amino acids or acidic amino acids, and the specificity of amino acid sequence is involved [21].
Trypsin	The long aliphatic and unbranched parts of the basic arginine and lysine side chains [20].
Pronase E	A mixture of exo- and endoproteases [37]. The release of other components that were associated with proteins [33].
Carbohydrates	
Iodate	Reacting with SH-groups, lipids and cysteine [38].
Periodate	Reacting with SH-groups, lipids and cysteine [38]. Cleaving C-C bonds in 1,2-diol and 1,2,3-triol compounds (e.g., carbohydrate components) to yield dialdehyde fragments and iodate by further oxidation and hydrolysis [39].
Lysozyme	Hydrolysis of β-1,4-glycosidic bonds (lysis of peptidoglycan layers) [40].
Carboxyl groups and ester groups	
Methanol	Substitution of carboxyl groups by ester groups. Enhancing surface hydrophobicity [41] and improving the electronegativity of cell surface [42].
Lipase	Ester groups [43].

### Acid and NaOH pre-treatments

The HCl pre-treatment induced the perturbation of the bacterial cell wall, which promoted or inhibited patulin adsorption (**Fig 4**) depending on the degree of destruction in the cell walls. Compared with acid pre-treatment, NaOH pre-treatment could neutralize acidic chemical groups of heat-inactivated LAB cells, and improve the electronegativity of cell surface (**Table 2**). It could explain why the NaOH pre-treatment only resulted in an enhancement of adsorption of patulin for the electrophilic properties of patulin [19]. The enhancement of patulin adsorption ability after NaOH pre-treatment was in agreement with a previous study on a treated yeast strain [14]. Moreover, Guo *et al*. [8] acknowledged the high ability of a NaOH pre-treated yeast strain to reduce patulin level in apple juice. Thus, the NaOH pre-treatment should be an effective and broadly available method to improve the adsorption ability of heat-inactivated LAB cells.

### Trypsin and pepsin pre-treatments

It is well documented that Asp 189, at the bottom of the active site pocket of trypsin, is the major determinant for the narrow specificity of trypsin [20]. The hydrophobic walls of the pocket create a favorable environment for the long aliphatic and unbranched parts of the basic arginine and lysine side chains [20]. It was clear that the pre-treatment with trypsin caused a significant decrease compared with the solvent control (*P* < 0.05) for all the tested strains. Hence, the adsorption sites should contain alkaline amino acids, which are the preferred substrates for trypsin.

Pepsin pre-treatment significantly enhanced the ability of LR-6133 and EF-21605 to adsorb patulin, suggesting that the substrates for pepsin are not the adsorption sites and more adsorption sites were exposed after pepsin pre-treatment. For the other strains, there was no effect compared with the solvent control pre-treatment (Type *l'*) (*P* > 0.05). The possible reason is that some ‘anti-susceptible amino-acids’ exist in the amino acid sequence of them [21].

### Methanol (Esterification) and lipase pre-treatments

After carboxyl group esterification, adsorption abilities of heat-inactivated cells of the six LAB strains were enhanced greatly (**Fig 4**). These results again differed from those from Guo *et al*. [13] who showed that methanol pre-treatment of heat-treated yeast caused a significant decrease on patulin adsorption. Our results indicated that ester groups could boost patulin adsorption more than carboxyl groups through the effects on hydrophobic interaction and electrostatic interaction (**Table 3**). Moreover, the importance of ester groups were further confirmed by the negative impact of lipase pre-treatment on the adsorption abilities of heat-inactivated cells of all the strains tested (**Fig 4**), which was not consistent with the report of Guo *et al*. [13]. Those authors showed that lipase pre-treatment did not significantly affect the ability of a heat-treated yeast strain to adsorb patulin, although the presence of the hydrophobic interaction in the patulin adsorption had been suggested by the report. Generally speaking, our studies indicate that methanol pre-treatment (esterification) should also be an effective and broadly available method to improve the patulin adsorption ability of heat-inactivated LAB cells. Moreover, it should have the high promotion and application value for the other mycotoxin adsorption processes. For example, a kind of chemically modified montmorillonite had a higher affinity to zearalenone for the increased hydrophobicity of the clay surface [22].

### Iodate, periodate, and lysozyme pre-treatments

Although the actions of periodate and iodate have close connection and similarities, they cause some different effects on microbial cells based on different anions (**Table 3**). Therefore, periodate oxidation analysis of carbohydrates often takes iodate treatment as the comparison. For all the tested strains, the results showed that the adsorption abilities were not significantly different between sodium iodate and sodium periodate pre-treated heat-inactivated cells (*P* > 0.05; **Fig 4**). Our results differ from those reported by Guo *et al*. [13] who showed that the pre-treatment of a heat-treated yeast strain with periodate caused a significant decrease on patulin adsorption compared with the iodate control. Additionally, the solvent control (Type *k′*) was added in this study, which has not been reported yet. For most of the strains, both of sodium iodate and sodium periodate pre-treatments significantly decreased the adsorption abilities compared with the solvent controls. The results implied that SH-groups, lipids or cysteine were more relevant to patulin adsorption than vicinal OH-groups in carbohydrate components or other components.

Lysozyme is often used for the lysis of peptidoglycan layers (**Table 3**). The adsorption efficiency of lysozyme pre-treated heat-inactivated cells was lower (for LC-21019, LB-20023, and EF-21605) than, or equivalent (for LR-6133, LR-6224, and EF-20420) to that of untreated heat-inactivated cells. This was not consistent with the observations that the pre-treated cells of LR-6133, LR-6224, EF-20420, and EF-21605 strains were significantly damaged; while for LC-21019 and LB-20023 the effects were more moderate (**Fig 3**).

Basically, our results suggested that although the peptidoglycan played a positive role in promoting patulin adsorption, the efficacy was not a principal factor, ignoring the function components which was typically coupled to, or embedded in the peptidoglycan. Our studies revealed that the physical structure of peptidoglycan was not a principal factor.

### Acetone, urea, and formaldehyde pre-treatments

In this study, the impacts of acetone, urea, and formaldehyde pre-treatments appeared to be strain specific. Thus, our results are not completely consistent with previous reports [13,14]. Our results could be explained in the following aspects to deepen the understanding and awareness of the adsorption mechanism. For acetone pre-treatment, the negative impacts of the pre-treatment was caused by the solubilization of non-covalently bound apolar glycolipids, while the positive impacts was caused by the increase of the adsorption sites for the changes of the membrane permeability, the intracellular structures, and the cell wall composition [23,24]; for urea pre-treatment, our results suggested that besides the negative impacts (**Table 2**), part of the effects may be similar to the NaOH pre-treatment, which promoted the adsorption of patulin; for formaldehyde pre-treatment (Eschweiler-Clarke methylation), the results could be explained by the fact that the pre-treatment can also facilitate or block protein-protein interactions (**Table 3**) which were strain-dependent and could influence the properties of patulin adsorption.

As can be seen in **Fig 5**, the adsorption abilities were almost maintained as pH was ≤ 4.0, whereas, when the pH value was raised from 4.0 to 6.0, the adsorption abilities were enhanced significantly. This finding is different with the results of other authors [7,15,17,18]. The results in our study can be explained by the electrophilic properties of patulin [19]. At low pH, the cell surface becomes more positively charged, reducing the attraction between heat-inactivated cells and patulin. In contrast, higher pH results in facilitation of the patulin adsorption, since the cell surface is more negatively charged. Therefore, our study indicated that the participation level of electrostatic attraction was very low in the adsorption phenomenon observed under low pH conditions (2.2 < pH ≤ 4). Conversely, the interpretation of the adsorption mechanism that occurred at 4 < pH < 6 should take the electrostatic attraction aspect into consideration.

The effects of the metal ions type and concentration on the patulin adsorption at pH 4 were slight and strain-dependent (**Fig 6**). According to the differences of surface charge among bacterial species and conclusions from the influence of pH on the adsorption, we posit that the adsorption mechanism that occurred at about pH 4 was strain-specific. In other words, besides hydrophobic interaction, at about pH 4 some adsorption processes had weak electrostatic attractions; other had weak electrostatic repulsions; yet some hardly had any electrostatic interaction. In addition, besides the influence on the electrostatic interaction between the adsorbent surface and the adsorbate, the increasing ionic concentration would further induce the variation of the hydrophobic interaction [25].

Although this work did not determine the total amount of patulin desorbed from cells-toxin complexes after washes and extractions, the results presented herein provided some useful information. As patulin was still released from the washed cell-patulin complexes, it confirmed that the adsorption of patulin was reversible, to a limited degree. Additionally, it is interesting to note that the strain (LB-20023, EF-20420, or EF-21605) which was always more efficient in patulin adsorption released lower proportion of patulin than the other strains studied here, indicating that the complex formed with LB-20023, EF-20420, or EF-21605 was more stable. It manifested that the adsorption involved weak non-covalent interactions, and a proportion of patulin which was strongly bound to the heat-inactivated cells was strain-specific.

As can be seen from our results, the adsorption performances of six LAB strains and most of the impacts of pre-treatments, simultaneous treatments, and post-treatments were strain-dependent. It provides some insights for the application through the results. Firstly, a cocktail of various LAB strains that adsorbs the patulin in different ways might be more effective to lower the level of patulin, which was seldom recorded in literatures. Secondly, the addition of very promising derived products of LAB strains might provide versatile tools of preventing mycotoxicosis. Thirdly, further intensive screening of LAB strains may allow patulin detection and removal to perform simultaneously, with low patulin-release ability.

As a future perspective, main applications of this research could be show in the apple industry because patulin poses a great threat to apples and their respective products [5]. During the production of apple juice, before using a rotary vacuum pre-coat filter, heat-inactivated LAB cells or their derived products could be more effective to remove patulin from apple juice as a kind of powdered materials. The reasons can be listed as follows. One study showed that depectinization, clarification, and filtration using a rotary vacuum pre-coat filter was found to be more effective than using ultrafiltration for the removal of patulin from apple juice [26]. Moreover, Bissessur *et al*. [27] showed that the removal of patulin depends mostly on bonding of the patulin to particulate material, which was removed in the clarification step.

In this study, key factors involved in the mechanism of patulin adsorption by heat-inactivated LAB strains were screened by various treatment methods and SEM. It showed that the physical structure of peptidoglycan was not a principal factor. Vicinal OH-groups and carboxyl groups were not significantly involved in patulin adsorption, and alkaline amino acids, thiol and ester compounds were important for the adsorption of patulin. Besides hydrophobic interaction, electrostatic interaction also participated in the patulin adsorption and it was enhanced with the increase of pH (4.0–6.0). NaOH and esterification pre-treatments could widely increase patulin adsorption abilities of heat-inactivated LAB strains. Lower proportion of patulin was released from the strain with higher adsorption ability. It should be noted that physical adsorption and chemical adsorption may proceed at the same time in the adsorption process of patulin; thus, further research is required in order to investigate the main pattern and type of patulin adsorption. This study can partly eliminate the limitations and issues reported in previous studies. Our study provides a better understanding of the adsorption mechanism of patulin by heat-inactivated LAB cells and provides some experimental and theoretical basis for the optimization and establishment of procedures of this technology.
